# Analysis of two mechanisms of telomere maintenance based on the theory of g-Networks and stochastic automata networks

**DOI:** 10.1186/s12864-020-06937-9

**Published:** 2020-09-09

**Authors:** Kyung Hyun Lee, Marek Kimmel

**Affiliations:** 1 Department of Statistics, Rice University, 6100 Main Street, Houston, 77057 TX USA; 2 Department of Systems Biology and Engineering Silesian University of Technology, Akademicka 16, Gliwice, 44-100 Poland

**Keywords:** Queueing network theory, G-networks, Stochastic automata networks, Correlation analysis, Gene regulatory networks, Telomeres

## Abstract

***:**

Background Telomeres, which are composed of repetitive nucleotide sequences at the end of chromosomes, behave as a division clock that measures replicative senescence. Under the normal physiological condition, telomeres shorten with each cell division, and cells use the telomere lengths to sense the number of divisions. Replicative senescence has been shown to occur at approximately 50–70 cell divisions, which is termed the Hayflick’s limit. However, in cancer cells telomere lengths are stabilized, thereby allowing continual cell replication by two known mechanisms: activation of telomerase and Alternative Lengthening of Telomeres (ALT). The connections between the two mechanisms are complicated and still poorly understood.

***:**

Results In this research, we propose that two different approaches, G-Networks and Stochastic Automata Networks, which are stochastic models motivated by queueing theory, are useful to identify a set of genes that play an important role in the state of interest and to infer their previously unknown correlation by obtaining both stationary and joint transient distributions of the given system. Our analysis using G-Network detects five *statistically significant* genes (CEBPA, FOXM1, E2F1, c-MYC, hTERT) with either mechanism, contrasted to normal cells. A new algorithm is introduced to show how the correlation between two genes of interest varies in the transient state according not only to each mechanism but also to each cell condition.

***:**

Conclusions This study expands our existing knowledge of genes associated with mechanisms of telomere maintenance and provides a platform to understand similarities and differences between telomerase and ALT in terms of the correlation between two genes in the system. This is particularly important because telomere dynamics plays a major role in many physiological and disease processes, including hematopoiesis.

## Background

The introduction of *Stochastic Automata Networks* (SANs) [1] has led researchers to explore an efficient solution of finite multi-component Markov chains of potentially very high dimension. The main idea of the SAN formalism is to employ Kronecker algebra operations for generating the *infinitesimal generator* (also known as an *intensity matrix* or *transition rate matrix*) of a Markov chain in a product form in order to ease the problem of dimensionality and the complexity of the vector-matrix product [2, 3]. Specifically, a compact representation using tensor product and sums of matrices of the form, which is formally proved in [1]

1 $$\begin{array}{*{20}l}  Q = \bigoplus_{i=1}^{N} L_{i} + \sum_{r = 1}^{R} \; \left(\; \bigotimes_{i=1}^{N} \; M_{r,i} + \bigotimes_{i=1}^{N} \; N_{r,i} \; \right), \end{array} $$

provides the transition rates of a time-continuous Markov chain of the type just described. Notation: *N* is the number of automata, *R* is the number of synchronizations, and *L*_*i*_ and *M*_*r*,*i*_ are matrices containing information about the local transitions and the effect of the synchronization *r* on the automaton *i*, respectively. *N*_*r*,*i*_ is the normalizing matrix of *M*_*r*,*i*_, and ⊗ and ⊕ denote, respectively, the (generalized) tensor product and tensor sum [4]. Notation will be discussed in detail in the following section.

For a decade after SAN introduction, many outstanding analytical results have been proved. For example, Plateau and Stewart [5] proved in year 2000 that a product-form steady-state distribution for SANs *without* synchronizations exists, as long as some numerical conditions related to local balance are satisfied. Prior to that, Boujdaine et al. in 1997 [6] considered a special class of SAN having *limited synchronization*, and proved a sufficient condition for existence of stationary distribution, by applying properties of Kronecker sum. Note that classic queuing networks such as Jackson’s networks and G-Networks with positive and negative customers [7] fall under this special class.

Most analyses and applications of SANs focus on finding steady-state distribution of queuing system models. To our knowledge, *correlation analysis* in SANs is rarely examined, although *correlation* quantifies the degree of inter-relatedness of two automata. It contributes to the understanding of how the association of two automata changes with time according to the state of interest, with the behavior of other automata in the system simultaneously considered. In the current study, we first examine the exact-form of an infinitesimal generator for G-Networks with positive and negative customers. Then it is applied to a gene regulatory network (GRN) having five genes related to the onset of cancer in order to show the time-dependent dynamics of transition rates and correlation between two particular genes of interest. Throughout this paper, our analysis is based on time-continuous Markov chains, but we note that most results are valid in the time-discrete case provided complications such as periodicity are excluded.

### Stochastic automata network

The SAN consists of a number of automata, which are correlated by synchronizing transitions. Each automaton has states and transitions. The state space of the system is the Cartesian product of the states of the automata [6]. There exist two types of transitions: local and synchronizing.

1. Transition in one automaton may initiate a new *simultaneous* transition in another automaton. Such transitions are collectively referred to as *synchronizing* transitions. In one synchronizing transition, two automata are paired. The first is called a *master* automaton; it affects the state of another automaton as its state changes. The second is called a *slave* automaton; it is affected by its *master* automaton [8].
2. In any given automaton, transitions not classified as synchronizing transitions are *local* transitions. Such transitions only change the state of one automaton.

In the SAN, two assumptions are made. First, the time to transition is *exponentially* distributed, and second, that a multidimensional Markov chain represents the SAN, although a particular automaton may not be Markovian itself. Then the (global) infinitesimal generator (the matrix of time derivatives at 0 of the transition probabilities), which reflects a change in transition probability from one state to another, is given by Eq. 1, with notation explained in Table1.

**Table 1:** Description of the notation in Eq. 1

Notation	Definition
*N*	Number of automata in the network
*K*	Number of states, where the state space of each automaton is {0,1,⋯,*K*−1}
*R* and *r*	Number of synchronizing events and an index of each event, respectively
*L*_*i*_	Local transition rate matrix (normalized) of the automaton *i*
*M*_*r*,*i*_	Effect matrix corresponding to the *r*^*th*^ synchronizing event acting on the automaton *i*
*N*_*r*,*i*_	Normalizing matrix needed for the tensor product of *M*_*r*,*i*_ to constitute a transition rate matrix

Specifically, each matrix in *Q* is defined as follows:

- *L*_*i*_ is a local normalized transition rate matrix of automaton *i*; thus
  $$\begin{array}{*{20}l} L_{i}[m,n] \geq 0 \;\; \text{if} \;\; m \neq n \;\;\; \text{and} \;\;\; \sum_{n = 1}^{N} L_{i}[m,n] = 0 \end{array} $$
- For each synchronizing transition, for all *i*∈{1,2,⋯,*N*},*M*_*r*,*i*_=*I*_*K*_, where *I*_*K*_ is an identity matrix of size *K*, except for the case when *r* is two indices corresponding to master and slave automaton. Therefore
  $$ {{{}\begin{aligned} \bigotimes_{i=1}^{N} \; M_{r,i} & = I_{K} \otimes \cdots \otimes I_{K} \otimes \left(D_{r, msr(r)} - \bar{D}_{r, msr(r)} \right) \\ & \;\;\;\;\; \otimes I_{K} \otimes \cdots \otimes I_{K} \otimes E_{r, sl(r)} \otimes I_{K} \otimes \cdots \otimes I_{K} \end{aligned}}} $$
  where *msr*(*r*) and *sl*(*r*) are indices for master automaton and slave automaton, respectively, in the *r*^*th*^ synchronization. Here, *D*_*r*,*msr*(*r*)_ denotes the transition *rate* matrix due to the *r*^*th*^ synchronizing event on the master automaton, and $\bar {D}_{r, msr(r)}$ is its diagonal matrix. *E*_*r*,*sl*(*r*)_, the transition *probability* matrix, is the effect of the *r*^*th*^ synchronizing event on the automaton *sl*(*r*).
  $${{\begin{aligned} D_{r,msr(r)} [m, n] & \geq 0 \; \text{if} \; m \neq n \; & \text{and} \; & \sum_{n=1}^{N} D_{r,msr(r)} [m, n] & = 0 \;, \; \forall \; m \\ E_{r, sl(r)}[m,n] & \geq 0 \; \forall \; m, n & \text{and} \; & \sum_{n = 1}^{N} E_{r, sl(r)}[m,n] & = 1 \;, \forall \; m \end{aligned}}} $$
- A normalizing matrix $\bigotimes _{i=1}^{N} \; N_{i, r}$ is a (*K*^*N*^×*K*^*N*^)-dimensional diagonal matrix, whose the *l*^*th*^ diagonal element is the negative sum of the *l*^*th*^ row of $\bigotimes _{i=1}^{N} \; M_{r,i} $.

### G-Networks with positive and negative customers

**Fig. 1:**
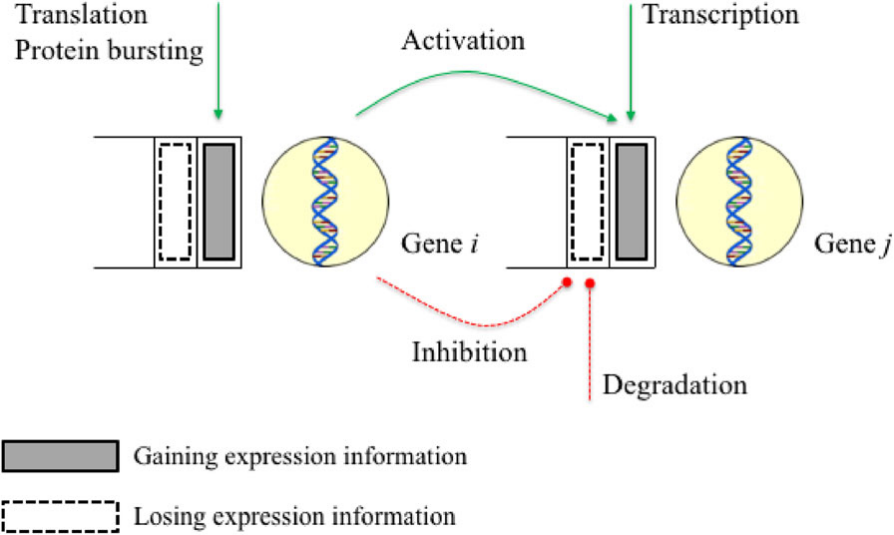
Four activities for gene regulation in a G-network model

Gelenbe- or G-Networks [9], devised by *Erol Gelenbe*, are Markovian stochastic models grounded in *queueing network theory* [10]. They were extensively applied to study probability models of various objects [11–18]. However they are particularly appropriate to construct GRNs, as they introduce a novel notion called a ‘*negative* customer’, which can be biologically interpreted as a *repression signal* [19]. G-Networks model the number of *positive* customers, which is *mRNA expression level* biologically, in an automaton or a *gene* having an infinite state space. *Positive* and *negative* customers *synchronously* move from a *master* gene to a *slave* gene within the system with transition probabilities, $p_{msr(r), sl(r)}^{+}$ and $p_{msr(r), sl(r)}^{-}$, respectively. It implies that there are two types of the synchronizing transition. Note that the behavior of positive customers identical to that of positive customers in Jackson networks [20]. In contrast, negative customers function distinctively as follows: they are not accumulated and instantaneously leave a queue after the completion of their tasks. If a negative customer arrives at a non-empty queue, it destroys one current positive customer. However if the queue is empty, then the negative customer disappears without *locally* affecting the queue. Both types of customers arrive at the *i*^*th*^ gene from outside of the system at rates $\lambda _{i}^{+}$ and $\lambda _{i}^{-}$, respectively. It is assumed that service disciplines are the same for all queue [21], and the service times for each queue *i* are independent and identically exponentially distributed with rates *μ*_*i*_∈(0,*∞*) for *i*=1,2,⋯,*N*. Figure 1 visually illustrates four activities of mRNA and/or protein molecules, and Table 2 suggests how the biological terms that refer to these activities can be identified with those used in G-Networks.

**Table 2:** Vocabulary and notation for application of G-Networks to Gene Regulatory Networks

Biology	G-Networks	Notation
Gene	Node where customers are stored	*i*
Signals/notch in the scale of the gene activity information [19]	Positive/negative customers	
mRNA expression level	The number of positive customers	*x*_*i*_
Translation / protein bursting	Arrival rate of positive customers from the outside of the system	$\lambda _{i}^{+}$
Degradation	Arrival rate of negative customers from the outside of the system	$\lambda _{i}^{-}$
Activation / transcription	Transition probabilities of positive customers	$p_{{ij}}^{+}$
Repression	Transition probabilities of negative customers	$p_{{ij}}^{-}$
Signals not influencing the gene activity	Customers that exit the system	*d*_*i*_
Protein-protein interaction	Service rate	*μ*_*i*_

The first and second columns contain biological and G-Networks terminologies of gene regulation, respectively. The third column includes the corresponding notations used in this study. The subscript *i* indicates the *i*th gene

G-Networks have an advantageous property of being analytically tractable because of the existence of product-form stationary distributions under the typical Markov Chain assumptions as shown in Eq. 2 [7, 22].

Let ***x***={*x*_1_,*x*_2_,⋯,*x*_*N*_} be the vector of non-negative integers representing the state of the network. Then the time-dependent vector, {***x***(*t*):*t*≥0}, is a continuous-time Markov chain, which satisfies the system of Chapman-Kolmogorov equations [23]. Element *x*_*i*_(*t*),1≤*i*≤*N*, of vector ***x***(*t*)=(*x*_1_(*t*),*x*_2_(*t*),⋯,*x*_*N*_(*t*)), is the number of customers (or the mRNA expression level) of gene *i* at time *t*. It has been proved that the joint steady-state distribution *π*(*x*) of ***x***(*t*), has the form of a product of stationary distributions of each queue,

2 $$\begin{array}{*{20}l}  \pi \left(x \right) = \lim\limits_{t \rightarrow \infty} P \left(\boldsymbol{X}(t) = \boldsymbol{x} \right) = \prod_{i=1}^{N} \left(1 - q_{i} \right) \cdot q_{i}^{x_{i}} \end{array} $$

each satisfying the balance equation of the G-Networks [23–25], i.e.

3 $$\begin{array}{*{20}l}  q_{i} \,=\, \lim\limits_{t \rightarrow \infty} P \left(x_{i}(t) > 0 \right) \,=\, \frac{\lambda_{i}^{+} + \sum_{j=1}^{N} q_{j} \cdot \mu_{j} \cdot p_{{ji}}^{+}}{\mu_{i} + \lambda_{i}^{-} + \sum_{j=1}^{N} q_{j} \cdot \mu_{j} \cdot p_{{ji}}^{-}} > 0, \end{array} $$

for *i*=1,2,⋯,*N*. It implies that the stationary probability for each positive recurrent state is expressed in the terms of a product of functions depending solely on the state of a single queue.

According to [8], matrices constituting the global infinitesimal generator *Q* are defined as follows in the terms of customer-related rates:

$${{\begin{aligned} L_{i} & = \lambda_{i}^{+} \left(Upp - I_{K}^{0} \right) + \mu_{i} d_{i} \left(Low-I_{1}^{0} \right) + \lambda_{i}^{-} \left(Low - I_{1}^{0} \right), \forall \; i \in \mathbb{N} \\ D_{r, msr(r)} & = \left\{\begin{array}{ll} & \mu_{i} \cdot \; p_{msr(r), sl(r)}^{+} \cdot \left(Low-I_{1}^{0} \right) \\ & \text{, if a customer leaves a master automaton} \\ & \text{and moves to a slave automaton as a} {positive} \text{ customer} \\ & \mu_{i} \cdot \; p_{msr(r), sl(r)}^{-} \cdot \left(Low-I_{1}^{0} \right) \\ & \text{, if a customer leaves a master automaton} \\ & \text{and moves to a slave automaton an as a } {negative} \text{ customer} \end{array}\right. \\ E_{r, sl(r)} & = \left\{\begin{array}{ll} \; Upp & \text{, if a } {positive} \text{ customer arrives at a slave automaton} \; sl(r) \\ \; Low & \text{, if a } {negative} \text{ customer arrives at a slave automaton} \; sl(r) \end{array}\right. \\ \bigotimes_{i=1}^{N} \; N_{r,i} & = \left\{\begin{array}{ll} I_{K} \otimes \cdots \otimes I_{K} \otimes \bar{D}_{r, msr(r)} \otimes I_{K} \otimes \cdots \otimes I_{K} \otimes I_{K}^{0} \otimes I_{K} \otimes \cdots \otimes I_{K} \\ \;\;\;\;\;\;\;\;\;\;\;\;\;\;\;\; \text{, if a slave automaton receives a positive customer} \\ I_{K} \otimes \cdots \otimes I_{K} \otimes \bar{D}_{r, msr(r)} \otimes I_{K} \otimes \cdots \otimes I_{K} \otimes I_{1}^{0} \otimes I_{K} \otimes \cdots \otimes I_{K} \\ \;\;\;\;\;\;\;\;\;\;\;\;\;\;\;\; \text{, if a slave automaton receives a negative customer} \end{array}\right. \end{aligned}}} $$

where

- *I* : Identity matrix
- *Upp* : Matrix with entries 0 except the main upper diagonal which is 1
- *Low* : Matrix with entries 0 except the main lower diagonal which is 1
- $I_{1}^{0}$ : Identity matrix except the first diagonal element which is 0
- $I_{K}^{0}$: Identity matrix except the *K*^*th*^ diagonal element which is 0

## Results

This section consists of two parts. The first part shows the mathematical result that derives the correlation between two genes utilizing Kronecker algebra. The second part contains the biological results based on the application of the mathematical result to the gene regulatory network associated with telomere biology.

### Mathematical result: derivation of joint transient distribution of two automata

If *Q* is the infinitesimal generator and *P*(*t*) is the transition probability matrix of a finite Markov chain, we can derive a set of differential equations called *Kolmogorov’s forward equations*, in the form of *P*^′^(*t*)=*P*(*t*)·*Q*. Solution of the forward equation is given in this case by the power series expansion that converges for any square matrix *Q*:

4 $$\begin{array}{*{20}l} P(t) = e^{Qt} = \sum_{n=0}^{\infty} \frac{\left(Qt \right)^{n}}{n!}. \end{array} $$

The resulting (1×*K*^*N*^)-dimensional time-dependent state probability vector at time *t* has the form

5 $$\begin{array}{*{20}l} \pi(t) =\pi(0) \cdot e^{Qt} \end{array} $$

where *π*(*t*) is a state probability vector at time *t* [26]. In order to find a joint state probability vector *π*_*i*,*j*_(*t*) of two automata, say *i* and *j*, we introduce two matrices *C*_*i*,*j*_ and $C^{*}_{i,j}$, which are (*K*^*N*^×*K*^2^)- and (*K*^2^×*K*^*N*^)-dimensional, respectively. After several steps of calculation, we obtain

6 $$\begin{array}{*{20}l}  \pi_{i,j}(t) & = \underbrace{\pi(t)}_{\text{\(1 \times K^{N}\)}} \cdot \underbrace{C_{i,j}}_{\text{\(K^{N} \times K^{2}\)}} \\ & \Rightarrow \; C^{*}_{i,j} \cdot C_{i,j} \cdot e^{Q_{i,j} \; t} = C^{*}_{i,j} \cdot e^{Q \; t} \cdot C_{i,j} \;, \end{array} $$

where, for all *i*≠*j*, *i*,*j*=1,2,⋯,*N*, *C*_*i*,*j*_ and $C^{*}_{i,j}$ are defined by

$${\begin{aligned} \underbrace{C_{i,j}}_{\text{\(K^{N} \times K^{2}\)}} = \bigotimes_{n=1}^{N} \; U_{i,j,n} \;\;\; &, \;\;\; U_{i,j,n} = \left\{\begin{array}{ll} \;\; I_{K} \;\; & \; \text{, if} \; n = i \; \text{or} \; n = j \\ \;\; 1_{K} \;\; & \; \text{, if} \; n \neq i \; \text{and} \; n \neq j \end{array}\right. \\ \underbrace{C^{*}_{i,j}}_{\text{\(K^{2} \times K^{N}\)}} = \bigotimes_{n=1}^{N} \; V_{i,j,n} \;\;\; &, \;\;\; V_{i,j,n} = \left\{\begin{array}{ll} \;\; I_{K} \;\; & \; \text{, if} \; n = i \; \text{or} \; n = j \\ \;\; 1^{T}_{K} \;\; & \; \text{, if} \; n \neq i \; \text{and} \; n \neq j \;\;\;. \end{array}\right. \end{aligned}} $$

Note that the (1×*K*)-dimensional matrix $1_{K}^{T} = \left [\begin {array}{llll} 1 & 1 & \cdots & 1 \end {array}\right ]$ indicates the matrix transpose of 1_*K*_. This results in

7 $$\begin{array}{*{20}l} C^{*}_{i,j} \cdot C_{i,j} = \left(\bigotimes_{n=1}^{N} \; V_{i,j,n} \right) \cdot \left(\bigotimes_{n=1}^{N} \; U_{i,j,n} \right) = K^{N-2} \cdot I_{K^{2}} \;\;, \end{array} $$

where

$$\begin{array}{*{20}l} V_{i,j,n} \cdot U_{i,j,n} = \left\{\begin{array}{ll} I_{K} \;\;\; & \;\;\; \text{, if} \; n = i \; \text{or} \; n = j \\ K \;\; (\text{a scalar}) \;\;\; & \;\;\; \text{, if} \; n \neq i \; \text{and} \; n \neq j \;\;. \end{array}\right. \end{array} $$

Therefore Eq. 6 finally becomes

8 $$\begin{array}{*{20}l}  e^{Q_{i,j} \cdot t} = \frac{1}{K^{N-2}} \cdot C^{*}_{i,j} \cdot e^{Q \; t} \cdot C_{i,j} \;\;. \end{array} $$

Equation 8 implies that a *time-dependent* joint state probability vector *π*_*i*,*j*_(*t*) can be easily calculated, once the infinitesimal generator, *Q*, of the entire system is obtained.

9 $$\begin{array}{*{20}l}  {}& \pi_{i,j}(t) \; = \; \pi_{i,j}(0) \cdot e^{Q_{i,j} \cdot t}  \\ & = \; \frac{1}{K^{N-2}} \cdot \pi_{i,j}(0) \cdot C^{*}_{i,j} \cdot e^{Q t} \cdot C_{i,j} \\ & = \; \left\{ p_{0, 0}(t), \; p_{0, 1}(t), \; p_{0, 2}(t) \cdots,\right. \\& \;\left. p_{K-1, K-2}(t), \; p_{K-1, K-1}(t) \right\} \end{array} $$

**Fig. 2:**
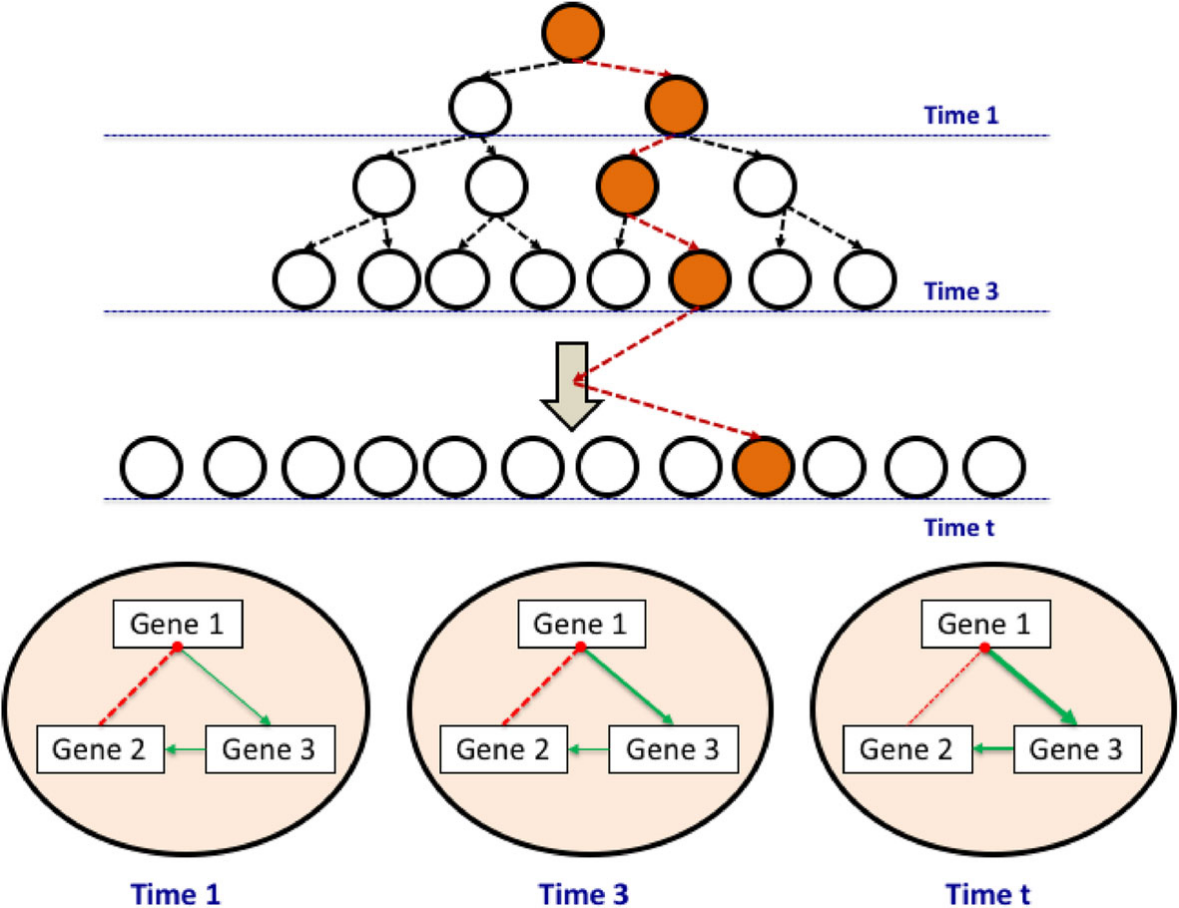
The correlation of a pair of genes. The correlation of a pair of genes can be either positive (green solid line) or negative (red dashed line). The thicker line represents the stronger correlation of two connected genes, while the thinner line indicates the weaker correlation of them

We can then form a (*K*×*K*)-dimensional table that describes the joint probabilities for each time point *t*. Based upon it, the association between two genes for all *t* is evaluated using the Pearson product-moment correlation coefficient [27]. It implies the correlation of a pair of genes in the regulatory system varies by each time (each generation of cells) as shown in Fig. 2.

### Application to the gene regulatory networks

#### GRNs and parameters from g-Networks

**Fig. 3:**
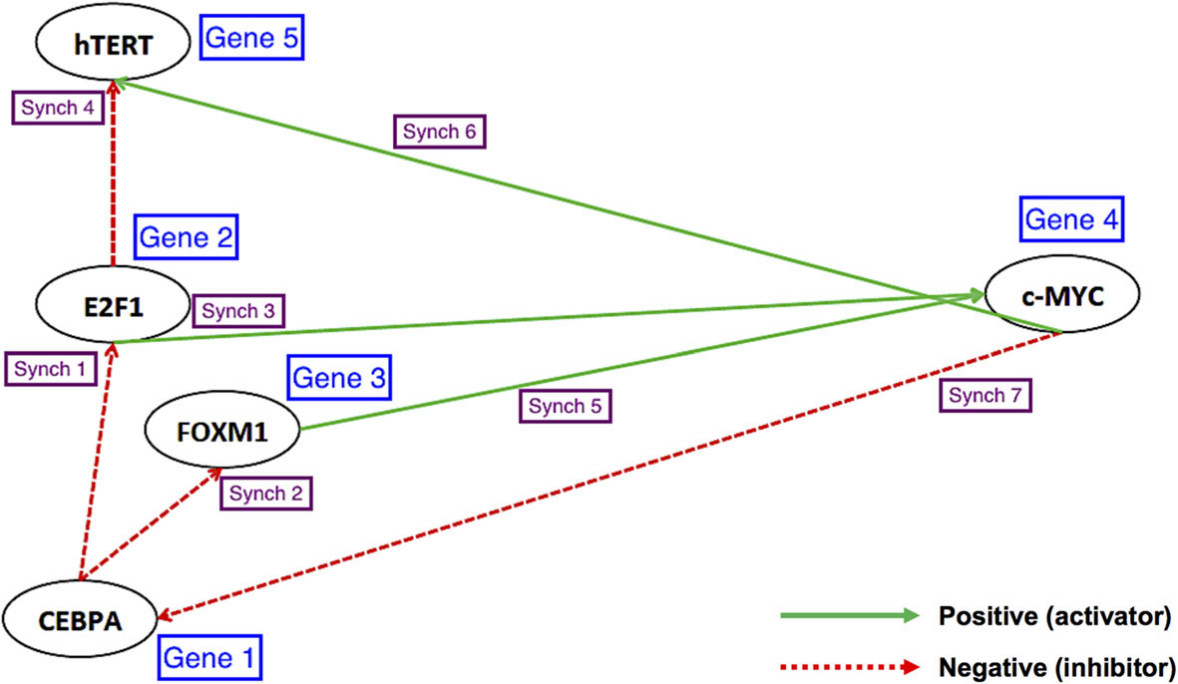
A network with 5 automata (genes) and 7 synchronizing transitions

In this research, we use a GRN provided in Fig. 3. It consists of 5 *statistically significant* genes associated with telomere maintenance, which are discovered by (modified) Abnormal Pathway Detection Algorithm (APDA) based on G-Networks [18]. (See Methods for detailed information on the modification). We explore the correlation between a pair of genes, e.g., CEBPA and hTERT, which is not apparent from Fig. 3. On the biology side, Kirwan et al. in 2009 [28] reported that mutations in hTERT within 4 of 20 families were identified in familial acute myeloid leukemia (AML). It is known that the mutation of CEBPA is one of the important factors in AML and its prognosis [29]. However the direct relationship between CEBPA and AML is still not satisfactorily understood. The joint state probability vector of two genes, which can be calculated from Eq. 9, is expected to show the flow of probabilities for the *K*^2^–tuples of mRNA expression levels at each fixed time *t*, and thereby illustrates the relationship between any two genes.

In this case, the number of automata is equal to *N*=5, while the number of synchronizing events is equal to *R*=7. We limit the number of gene expression levels to *K*=7 for two reasons. The first is that, because the data are normalized, there are few genes with a mRNA expression level of 7 or higher. Furthermore, the memory limitations enforce it. In this paper, we assume that pairs of genes are highly positively correlated at the initial time point; that is

$$\begin{array}{*{20}l} p_{{ij}}(0) = {\left[\begin{array}{lllllll} \frac{5}{47} & \frac{1}{47} & 0 & 0 & 0 & 0 & 0 \\ \frac{1}{47} & \frac{5}{47} & \frac{1}{47} & 0 & 0 & 0 & 0 \\ 0 & \frac{1}{47} & \frac{5}{47} & \frac{1}{47} & 0 & 0 & 0\\ 0 & 0 & \frac{1}{47} & \frac{5}{47} & \frac{1}{47} & 0 & 0 \\ 0 & 0 & 0 & \frac{1}{47} & \frac{5}{47} & \frac{1}{47} & 0 \\ 0 & 0 & 0 & 0 & \frac{1}{47} & \frac{5}{47} & \frac{1}{47} \\ 0 & 0 & 0 & 0 & 0 & \frac{1}{47} & \frac{5}{47} \\ \end{array}\right]} \end{array} $$

Note that this matrix needs to be transformed into a (1×*K*^2^)-dimensional joint state probability vector as *π*_*ij*_(*t*) in Eq. 9. Tables 3 and 4 exhibit the values of parameters that are required to build the infinitesimal generator *Q*. They are estimated and optimized according to the APDA [18] based on G-Networks. Specifically, values in Table 3 allow to calculate a joint probability vector *π*_*i*,*j*_(*t*), under the normal condition, with the Table 4 contains information for malignant (ALT or active telomerase) cells. The three families of parameters, $\lambda _{i}^{+}, \lambda _{i}^{-}$ and *μ*_*i*_ for all *i*, are identical for both normal and ALT cells, while transition probabilities, $p_{{ij}}^{+}, p_{{ij}}^{-}$ and *d*_*i*_, are different between the cell types. Transition probabilities are deciding factors in correlation coefficients based on Eq. 9 and the rates of convergence to stationarity in G-Networks.

**Table 3:** Values of the parameters needed to determine the infinitesimal generator (*Q*) using 4 *normal* cell lines, where $d_{i} = 1- \sum _{j=1}^{N} \left (p_{{ij}}^{+} + p_{{ij}}^{-}\right)$

	Translation ($\lambda _{i}^{+}$)	Degradation ($\lambda _{i}^{-}$)	*μ*_*i*_	*d*_*i*_	
CEBPA	4	1.18489	3	0.333	
E2F1	4	1.79118	3	0.333	
FOXM1	4	2.78510	2	0.5	
c-MYC	5	6.70809	3	0.333	
hTERT	5	6.07682	1	1	
Normal: Activation/transcription processes ($p_{{ij}}^{+}$)					
	CEBPA	E2F1	FOXM1	c-MYC	hTERT
CEBPA	0	0	0	0	0
E2F1	0	0	0	0.333	0
FOXM1	0	0	0	0.5	0
c-MYC	0	0	0	0	0.333
hTERT	0	0	0	0	0
Normal: Repression process ($p_{{ij}}^{-}$)					
	CEBPA	E2F1	FOXM1	c-MYC	hTERT
CEBPA	0	0.333	0.333	0	0
E2F1	0	0	0	0	0.333
FOXM1	0	0	0	0	0
c-MYC	0.333	0	0	0	0
hTERT	0	0	0	0	0

The “service” (or firing) rate of gene *i*, denoted by *μ*_*i*_, represents the protein–protein interactions, e.g., phosphorylation and ubiquitination. Gene *i* activates and inhibits gene *j* with probability $p_{{ij}}^{+}$ and $p_{{ij}}^{-}$, respectively. Genes in the rows correspond to the “starting” genes, while those in columns to the ending genes

**Table 4:** Values of the parameters that are needed to determine the infinitesimal generator (*Q*) using 18 ALT cell lines

	Translation ($\lambda _{i}^{+}$)	Degradation ($\lambda _{i}^{-}$)	*μ*_*i*_	*d*_*i*_	
CEBPA	4	1.18489	3	0.5433	
E2F1	4	1.79118	3	0.1258	
FOXM1	4	2.78510	2	0.2417	
c-MYC	5	6.70809	3	0.0280	
hTERT	5	6.07682	1	1	
ALT: Activation/transcription processes ($p_{{ij}}^{+}$)					
	CEBPA	E2F1	FOXM1	c-MYC	hTERT
CEBPA	0	0	0	0	0
E2F1	0	0	0	0.5574	0
FOXM1	0	0	0	0.7583	0
c-MYC	0	0	0	0	0.3855
hTERT	0	0	0	0	0
ALT: Repression process ($p_{{ij}}^{-}$)					
	CEBPA	E2F1	FOXM1	c-MYC	hTERT
CEBPA	0	0.2268	0.2230	0	0
E2F1	0	0	0	0	0.3168
FOXM1	0	0	0	0	0
c-MYC	0.5865	0	0	0	0
hTERT	0	0	0	0	0

The definitions and representations of $d_{i}, \mu _{i}, p_{{ij}}^{+}$ and $p_{{ij}}^{-}$ are the same as in Table 3

#### Simulation study using estimated parameters

Estimated and/or assumed values of the parameters listed in Tables 3 and 4 determine the infinitesimal generator, *Q*, and the steady-state distribution of each gene. Developed on the global balance equation of G-Networks [23] and the *exponentially-distributed* holding times of Markov chains [30], the *empirical cumulative density function* (ECDF) was exploited to confirm that parameter estimation via the APDA is appropriate aligning with the theory of G-Networks. We compare the ECDF of simulated data utilizing values in Tables 3 and 4 and the theoretical cumulative density function (CDF) based on *q*_*i*_ in Eq. 3. The algorithm for the simulation is explained in detail in Methods.

The ECDF, usually denoted by $\hat {F}_{n} (x)$, is associated with cumulative frequency of observations (empirical sampled data). It is a non-parametric estimator of the underlying CDF of a random variable of interest and is formally defined as follows:

$$\begin{array}{*{20}l} \hat{F}_{n} (x) = \hat{P}_{n} \left(X \leq x \right) = \frac{1}{n} \cdot \sum_{i=1}^{n} \; \mathbbm{1}_{\left[ x_{i} \leq x \right]} \;\;, \end{array} $$

where $\mathbb{1}_{\left [ \cdot \right ]}$ is an indicator function. That is, the ECDF is a step function that increases by $\frac {1}{n}$ at each datum. The stationary probability distribution of each gene in G-Networks is in the geometric product-form as stated in Eq. 2, which results in the following tail of the theoretical CDF, *T*(*x*_*i*_):

10 $$\begin{array}{*{20}l}  T \left(x_{i} \right) = 1 - F \left(x_{i} \right) = 1 - \left(1 - q_{i}^{x_{i} + 1} \right) = q_{i}^{x_{i} + 1} \;\;. \end{array} $$

**Fig. 4:**
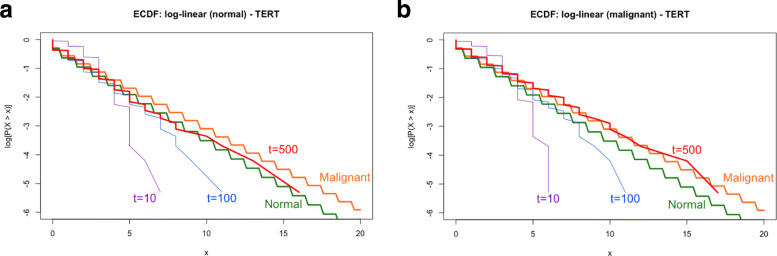
ECDF plots of hTERT under the normal condition (top) and ALT (malignant) condition (bottom). Orange and green straight and thick lines represent theoretical (logarithmically transformed) CDF of normal and ALT conditions, respectively. Purple, blue (thin) and red (thick) lines serve as the logarithmically transformed ECDF of hTERT after 10 steps, 100 steps and 500 steps, respectively

Figure 4 (and Fig. 9) illustrates how the ECDF of hTERT utilizing estimated/assumed values of the parameters (Tables 3 and 4) converges to the theoretical CDF regardless of cell types. Similar patterns are obtained for all 5 statistically significant genes in this research.

### Correlation between a pair of genes

**Fig. 5:**
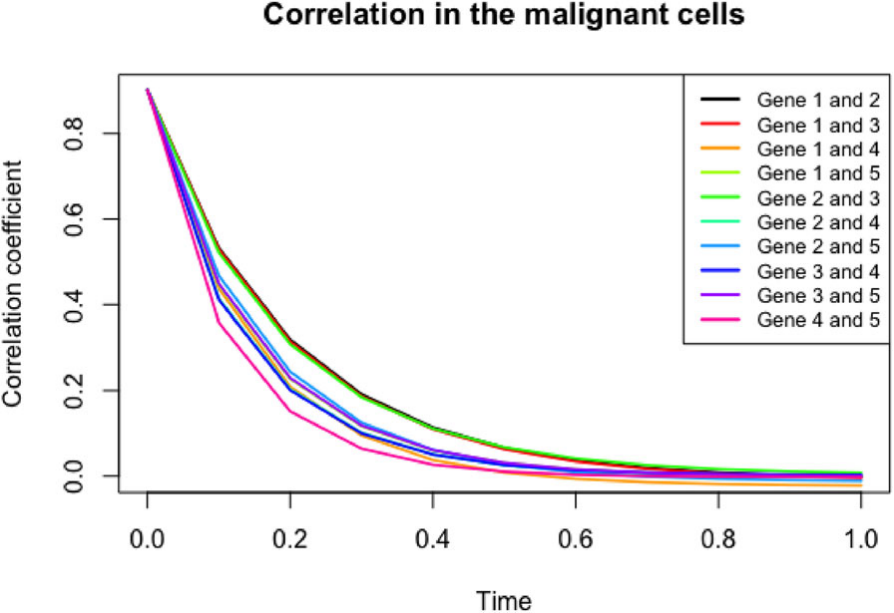
Trend of correlation between each pair of genes in the system over time in ALT (malignant) cells. The general relationship of two genes in the system is similar regardless of the mechanism by which telomeres maintain their sufficient lengths. However, within the same condition of cells, the magnitude of correlation and the speed to the steady state vary by interactions

We discuss the similarities or differences in the correlation of two genes between normal and malignant cells with either active telomerase or ALT. Figure 5 based on Eq. 9 illustrates the following:

- First, Fig. 5 demonstrates the change of correlation between a pair of genes in the given system over time in normal cells and ALT (malignant) cells. We can then infer both the trend of correlation between a pair of genes and the speed to the transient state, *i.e.*, before it reaches a steady state. Due to the product-form stationary probability distribution of network state in the theory of G-Networks, the correlation coefficient becomes 0 when the system becomes stationary [31]. Assuming that the pair of genes are positively correlated at time 0, the positive correlation persists over time in both types of cells (normal and ALT) and practically reaches 0 (the steady state) approximately at *t*=0.8. As shown in the figure, the patterns of correlation between each pair of genes do not noticeably differ among the types of cells (normal or malignant with either ALT or active telomerase shown), although they are dissimilar from one pair to another within the same type of cells. For example, the correlation between CEBPA and FOXM1 in a transient state is stronger than that of c-Myc and hTERT, and it reaches a steady state more slowly in all cell types.

**Fig. 6:**
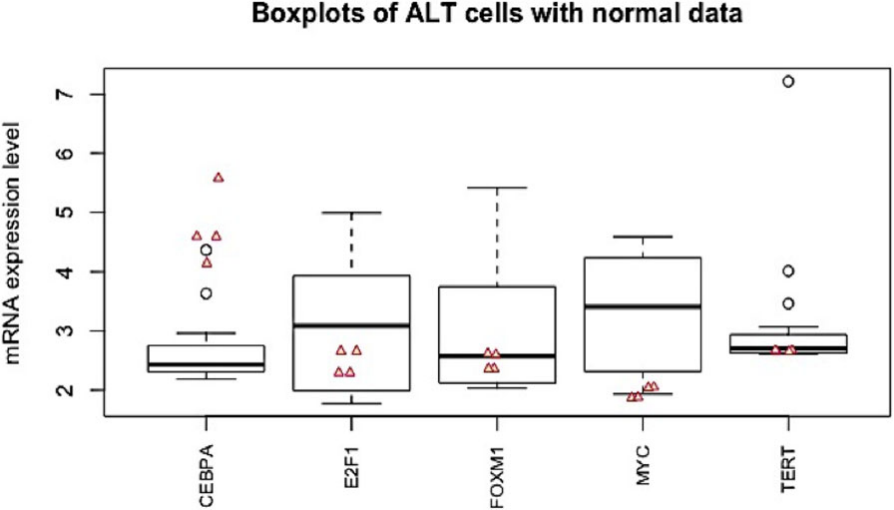
A box plot displaying the *five number summary* of the five significant genes in ALT cells. Red triangle dots represent the mRNA expression levels of the corresponding gene in 4 normal cells
- Second, Eq. 9 uncovers the interactions between (1) CEBPA and hTERT, (2) FOXM1 and hTERT and (3) FOXM1 and E2F1, which are unknown in Fig. 3. The pattern in Fig. 5, which is analogous to Fig. 7, delineates that the relationship of the three pairs of genes is similar regardless of the mechanisms by which telomeres maintain their sufficient lengths. However, within the same condition of cells, the magnitude of correlation and the speed to the steady state vary by interactions. It is supported by the interaction of CEBPA and hTERT, which is relatively weaker than that of other pairs, even though its trend over time is indistinguishable by the cell types.

**Fig. 7:**
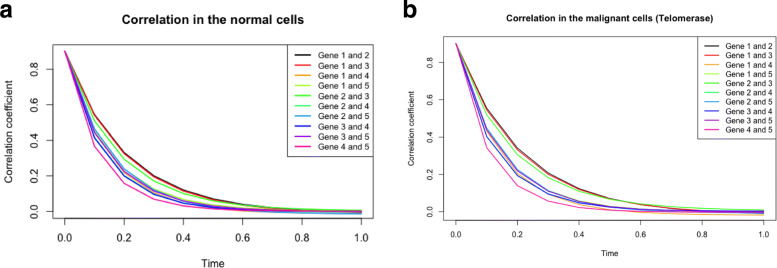
Trend of correlation between a pair of genes in the system over time in (top) normal cells and (bottom) in malignant cells from 16 telomerase-positive cell lines

**Fig. 8:**
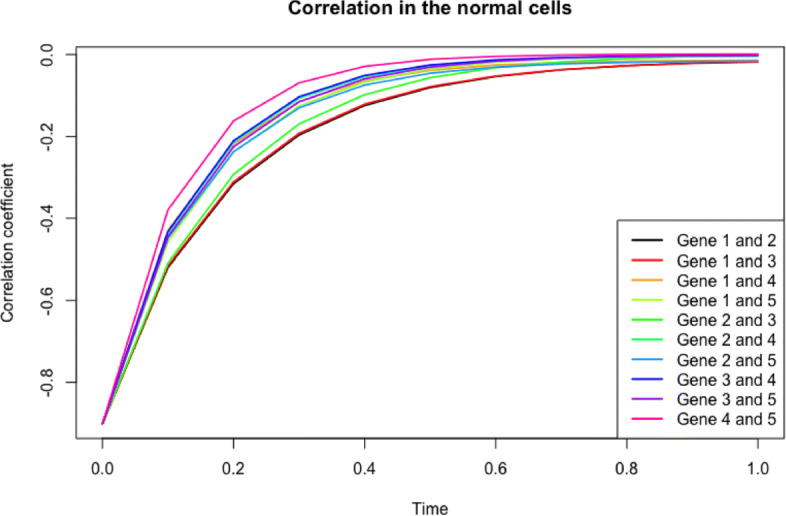
Trend of correlation between a pair of genes in the system over time in normal cells with the assumption that a pair of genes are initially negatively correlated

Note that if we conversely assume that a pair of genes are highly negatively correlated at the initial time point, then the negative correlation prevails over time as shown in Fig. 8.

**Fig. 9:**
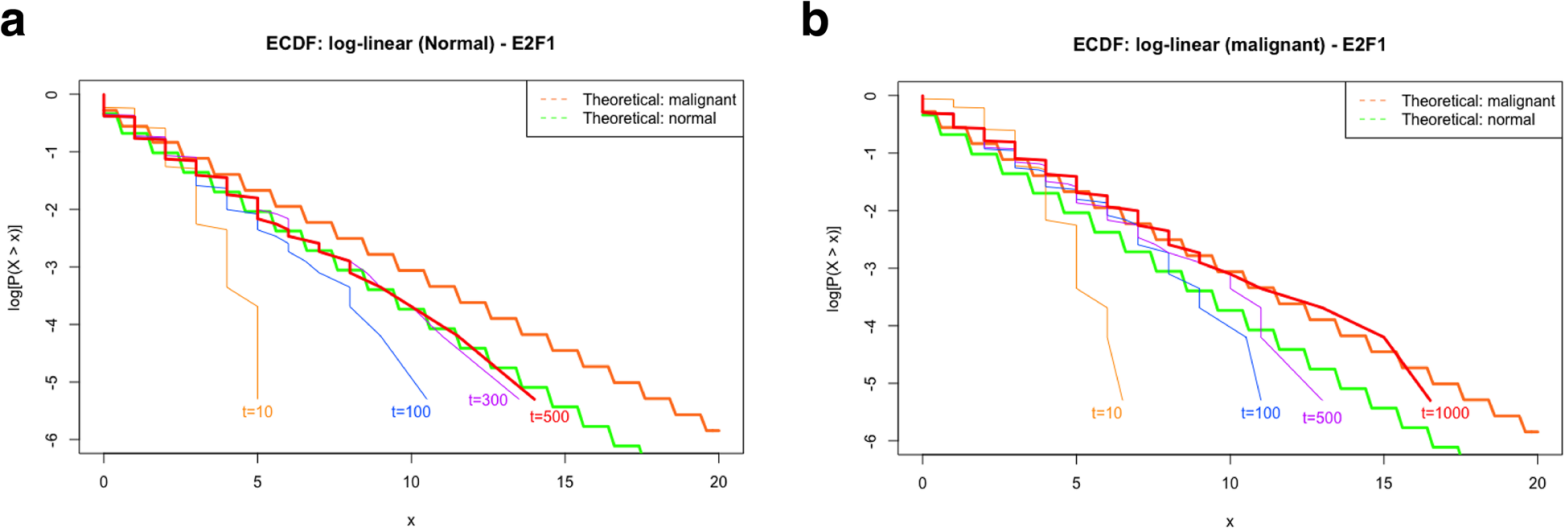
ECDF plots of E2F1 under the normal condition (top) and ALT (malignant) condition (bottom). Orange and green straight and thick lines represent theoretical (logarithmically transformed) CDF of normal and ALT conditions, respectively. Top: Orange, blue, purple, red (thick) lines serves as the logarithmically transformed ECDF of E2F1 after 10 steps, 100 steps, 300 steps and 500 steps, respectively. Bottom: Orange, blue, purple, red (thick) lines serves as the logarithmically transformed ECDF of E2F1 after 10 steps, 100 steps, 500 steps and 1000 steps, respectively

## Discussion

One important advantage of using mathematical modeling to understand dynamics of a biological system is that the model can be a cost-effective and time-saving supplement or even a substitute for laboratory experiments in which patterns of the system are delicate and complex. Especially the stochastic approach is useful to quantify the role of fluctuations in the behavior of the system of interest. In this paper, we propose that a stochastic paradigm involving *G-Networks* and *Stochastic Automata Networks* both stemming from queuing theory, can contribute to the analysis of similarities and differences between telomerase activation and ALT using genes related to telomere maintenance. However, there are the following two caveats.

- First, correlation coefficient, whose sign relies upon the initial joint state probability vector *π*_*ij*_(0) in our example, evaluates the strength of the evidence for a relationship of two genes but does not determine causal relationships. In other words, even if a positive correlation between CEBPA and hTERT in ALT cells is revealed, it is not known which gene activates another one.
- Second, the dimension of the infinitesimal generator, *Q*, which is a function of tensor products, can be enormously large, with the corresponding matrix being sparse depending on the number of states of each queue. The state space used in this research is relatively small. Computations in larger dimensions can be made practical by using Compressed Sparse Row and a number of methods for the approximation of the matrix exponential [32] such as Krylov-type techniques.

## Conclusion

In spite of the caveats discussed in the previous section, the APDA based on G-Networks initially introduced in [18] can suggest a set of *statistically significant* genes (CEBPA, FOXM1, E2F1, c-MYC, hTERT) in cells with either telomerase or ALT compared to normal cells from preexisting GRNs. We further confirmed that the APDA satisfactorily estimates the parameters, which decide the rates of convergence to stationarity as the ECDF of the simulated data using the estimated parameters approaches to theoretical CDF using *q*_*i*∈{1,2,⋯,*N*}_ as time progresses. Further, our correlation analysis based on SANs helps to infer the link between genes in various conditions that has not yet been discovered through experiments. We prove that our mathematical expression (Eq. 9) allows analytically calculating the correlation coefficients between any pair of genes in the given system at every time point and compare them in various types of cells for an order relation. Specifically in our example, the trend of correlation among genes in the system is not influenced by the mechanism (telomerase *vs*. ALT) by which telomeres maintain their sufficient lengths, even though the trend differs from one pair to another within the same type of cells. To conclude, this study provides a platform to detect significant genes and infer the previously unknown connection among them by applying modeling techniques borrowed from queueing theory. This is particularly important because telomere dynamics plays a major role in many physiological and disease processes, including hematopoiesis.

## Methods

### Detailed proof of Eq. 9

If *Q* is the infinitesimal generator and *P*(*t*) is the transition probability matrix of a finite Markov chain, we can derive a set of differential equations called *Kolmogorov’s forward equations*, in the form of *P*^′^(*t*)=*P*(*t*)·*Q*

$$\begin{array}{*{20}l} \frac{d}{dt} P(t) \equiv P^{\prime}(t) = P(t) \cdot Q \;. \end{array} $$

*P*(0) initial condition is *P*(0)=*I*, where *I* is an identity matrix having the same dimension as *Q* [33], consistent with

$$\begin{array}{*{20}l} p_{{ij}}(0) = \left\{\begin{array}{ll} P \left(\; X(0) = i \; | \; X(0) = i \; \right) = 1 \;\;\; &\text{, if} \; i = j \\ P \left(\; X(0) = j \; | \; X(0) = i \; \right) = 0 \;\;\; &\text{, if} \; i \neq j \end{array}\right. \end{array} $$

Solution of the forward equation for finite Markov chains is given by the power series expansion that always converges for any square matrix *Q*:

11 $$\begin{array}{*{20}l} P(t) & = e^{Qt} && = \sum_{n=0}^{\infty} \frac{\left(Qt \right)^{n}}{n!} \\ & && = I + Qt + \frac{\left(Qt \right)^{2}}{2!} + \frac{\left(Qt \right)^{3}}{3!} + \frac{\left(Qt \right)^{4}}{4!} \cdots  \\ & && \approx I + Qt + \frac{\left(Qt \right)^{2}}{2!} + \frac{\left(Qt \right)^{3}}{3!} + \|H(t)\|.  \end{array} $$

where ||·|| denotes *l*_1_-norm, and *H*(*t*) is is a linear operator with || *H*(*t*) ||=*o*(*t*), as *t*→0. When the infinitesimal generator, *Q*, is irreducible, its transition probability matrix, *P*(*t*) defined in Eq. 11, is strictly positive for each time *t*>0. It signifies that as the trajectory length *N* approaches to the limit, at least one transition between any pair of states will *almost surely* happen without regard to the sparsity of *Q* [34]. Note that 0=*π*·*Q* are a set of |*S*| linear equations, which is usually used to detect the stationary distribution *π*, if such one exists [26].

A formal solution to the time dependent state probability vector is defined as

12 $$\begin{array}{*{20}l}  \pi(t) = \underbrace{\pi(0)}_{\text{\(1 \times K^{N} \)}} \cdot \underbrace{e^{Qt}}_{\text{\(K^{N} \times K^{N} \)}} \end{array} $$

where *π*(*t*) is a state probability vector at time *t* [35, 36]. In order to find a joint state probability vector *π*_*i*,*j*_(*t*) of two automata, say *i* and *j*, we introduce two matrices called *C*_*i*,*j*_ and $C^{*}_{i,j}$, which are (*K*^*N*^×*K*^2^)- and (*K*^2^×*K*^*N*^)-dimensional matrices, respectively. Then based on Eq. 12, we achieve

13 $$\begin{array}{*{20}l} \begin{aligned} & &\pi_{i,j}(t) & = \underbrace{\pi(t)}_{\text{\(1 \times K^{N}\)}} \cdot \underbrace{C_{i,j}}_{\text{\(K^{N} \times K^{2}\)}} \\ & \Rightarrow \;\;\; & \pi_{i;j}(0) \cdot e^{Q_{i,j} \; t} & = \pi(0) \cdot e^{Q \; t} \cdot C_{i,j} \\ & \Rightarrow \;\; & \pi(0) \cdot C_{i,j} \cdot e^{Q_{i,j} \; t} & = \pi(0) \cdot e^{Q \; t} \cdot C_{i,j} \\ & \Rightarrow \;\; & C_{i,j} \cdot e^{Q_{i,j} \; t} & = e^{Q \; t} \cdot C_{i,j} \\ & \Rightarrow \;\; & C^{*}_{i,j} \cdot C_{i,j} \cdot e^{Q_{i,j} \; t} & = C^{*}_{i,j} \cdot e^{Q \; t} \cdot C_{i,j} \end{aligned} \end{array} $$

Here, *C*_*i*,*j*_ and $C^{*}_{i,j}$ are defined as follows respectively:

$${{\begin{aligned} \underbrace{C_{i,j}}_{\text{\(K^{N} \times K^{2}\)}} = \bigotimes_{n=1}^{N} \; U_{i,j,n} \;\;\;, \; \forall \; i \neq j \;, \; i = 1, 2, \cdots, N \;, \; j = 1, 2, \cdots, N \end{aligned}}} $$

where

$$\begin{array}{*{20}l} U_{i,j,n} = \left\{\begin{array}{ll} \;\; I_{K} \;\;\; & \;\;\; \text{, if} \; n = i \; \text{or} \; n = j \\ \;\; 1_{K} \;\;\; & \;\;\; \text{, if} \; n \neq i \; \text{and} \; n \neq j \end{array}\right. \end{array} $$

and

$${{\begin{aligned} \underbrace{C^{*}_{i,j}}_{\text{\(K^{2} \times K^{N}\)}} = \bigotimes_{n=1}^{N} \; V_{i,j,n} \;\;\;, \; \forall \; i \neq j \;, \; i = 1, 2, \cdots, N \;, \; j = 1, 2, \cdots, N \end{aligned}}} $$

where

$$\begin{array}{*{20}l} V_{i,j,n} = \left\{\begin{array}{ll} \;\; I_{K} \;\;\; & \;\;\; \text{, if} \; n = i \; \text{or} \; n = j \\ \;\; 1^{T}_{K} \;\;\; & \;\;\; \text{, if} \; n \neq i \; \text{and} \; n \neq j \;\;\;. \end{array}\right. \end{array} $$

Note that $1_{K}^{T}$ indicates the matrix transpose of 1_*K*_, and we have

$$\begin{array}{*{20}l} \underbrace{1_{K}}_{\text{\(K \times 1\)}} & = \left[\begin{array}{l} 1 \\ 1 \\ \vdots \\ 1 \end{array}\right] \;\;\;\;\; \text{and} \;\;\;\;\; \underbrace{1_{K}^{T}}_{\text{\(1 \times K\)}}= \begin{bmatrix} 1 & 1 & \cdots & 1 \end{bmatrix}. \end{array} $$

This results in

$$\begin{array}{*{20}l} C^{*}_{i,j} \cdot C_{i,j} & = \left(\bigotimes_{n=1}^{N} \; V_{i,j,n} \right) \cdot \left(\bigotimes_{n=1}^{N} \; U_{i,j,n} \right) \\ & = \bigotimes_{n=1}^{N} \; V_{i,j,n} \cdot U_{i,j,n} \\ & = K^{N-2} \cdot I_{K^{2}} \end{array} $$

where

$$\begin{array}{*{20}l} V_{i,j,n} \cdot U_{i,j,n} = \left\{\begin{array}{ll} I_{K} \;\;\; & \;\;\; \text{, if} \; n = i \; \text{or} \; n = j \\ K \;\; (\text{a scalar}) \;\;\; & \;\;\; \text{, if} \; n \neq i \; \text{and} \; n \neq j \end{array}\right. \end{array} $$

Therefore Eq. 13 finally becomes

14 $$\begin{array}{*{20}l} \begin{aligned}  & K^{N-2} \cdot I_{K^{2}} \cdot e^{Q_{i,j} \cdot t} && = C^{*}_{i,j} \cdot e^{Q \; t} \cdot C_{i,j} \\ \Rightarrow \;\;\; & e^{Q_{i,j} \cdot t} && = \frac{1}{K^{N-2}} \cdot C^{*}_{i,j} \cdot e^{Q \; t} \cdot C_{i,j} \end{aligned} \end{array} $$

Equation 14 implies that a *time-dependent* joint state probability vector *π*_*i*,*j*_(*t*) can be easily calculated, once the (global) infinitesimal generator of the entire system is obtained.

15 $$\begin{array}{*{20}l} \pi_{i,j}(t) & \; = \; \underbrace{\pi_{i,j}(0)}_{\text{\(1 \times K^{2}\)}} \cdot \underbrace{e^{Q_{i,j} \cdot t}}_{\text{\(K^{2} \times K^{2}\)}} \; = \; \frac{1}{K^{N-2}} \cdot \pi_{i,j}(0) \cdot C^{*}_{i,j} \cdot e^{Q t} \cdot C_{i,j} \end{array} $$

Once *π*_*i*,*j*_(*t*) of two genes is obtained, we then can form a (*K*×*K*)-dimensional contingency table that describes the joint probability for each time point *t*. Based upon it, the association between two genes for all *t* is evaluated using the Pearson product-moment correlation coefficient.

### Application to telomere maintenance

#### Data description

Gene expression data (GSE14533) were obtained from the National Center for Biotechnology Information Gene Expression Omnibus (http://www.ncbi.nlm.nih.gov/geo/). Data includes expression gene levels of 3 types of cells: 18 ALT cell lines, 16 telomerase-positive cell lines and 4 normal (fibroblast) cell lines. Initially, the gene expression levels of 29 genes (hTERT, CRY2, FOXM1, c-MYC, SOCS1, AHNAK, HDAC5, TK1, S100A4, LMO4, PRKD1, PER2, PRKCA, CEBPA, E2F1, RBPJ, TOMM20, AIP, EHD1, TGOLN2, TTC17, LAMP2, ATP5D, ADGRL1, LASP1, RPRD1A, HNRNPA3, KAT2A, STK24) were extracted from the data according to [37]. However, only 5 *significant* genes (CEBPA, E2F1, FOXM1, c-MYC, hTERT) (connected to *statistically significant* edges) in either telomerase-positive or ALT cells compared to the normal cells, discovered by APDA based on G-Networks [18], have been included in this research. Note that the data were first normalized to the 50^*th*^ percentile to guarantee the identical medians across all samples. Then they were normalized again and scaled with mean 3 and variance 1 [18, 38]. Table 5 and Fig. 6 outline the data in detail.

**Table 5:** Mean mRNA expression levels (normalized and scaled) of genes in normal and ALT cells

	Normal	ALT	Mean diff.	*P*-value
CEBPA (Gene 1)	4.724	2.617	2.107	0.003*
E2F1 (Gene 2)	2.475	3.117	-0.642	0.029*
FOXM1 (Gene 3)	2.484	3.115	-0.631	0.027*
c-MYC (Gene 4)	1.957	3.232	-1.275	0.000*
hTERT (Gene 5)	2.662	3.075	-0.414	0.128

*P*-values were obtained from the Student’s t–test based on the mean differences of the expression levels. An asterisk indicates statistical significance of *p*<0.05

### Modification of the aPDA

We adopt APDA introduced originally in [18], but slight modification of assumptions on $\lambda _{i}^{+}$ and $\lambda _{i}^{-}$ is added in this research as follows:

- Define *initiating* genes as genes which do not receive any customer from other genes but send a customer to others within the system.
- The arrival rate of negative customers from the external system, denoted by $\lambda _{i}^{-}$, of the *initiating* genes is assumed to be 0. This assumption enables the initiating genes to maintain their customers; thereby to be consistently activated.
- The arrival rate of positive customers from the external system is defined as follows:
  $${{\begin{aligned} \lambda_{i}^{+} = \left\{\begin{array}{ll} D_{i}^{\; in} + 3 \;\;\;\;\; &, \;\;\; \text{if} \;\; i \; \in \; \left\{ \text{Indices of non-initiating genes} \right\} \\ \frac{\bar{x}_{i}}{\bar{x}_{i} + 1} \times \mu_{i} \;\;\;\;\; &, \;\;\; \text{if} \;\; i \in \left\{ \text{Indices of initiating genes} \right\} \end{array}\right. \end{aligned}}} $$
  where $\bar {x}_{i}$ is the average mRNA expression level of the gene *i* in normal cell lines. Here, $D_{i}^{\; in}$ represents the *in-degree* of gene *i*, which is the number of edges incoming to node *i*. The purpose of adding 3 to the non-initiating genes is to prevent the numerator of *q*_*i*_ from becoming 0. The definition of $\lambda _{i}^{+}$ for the *initiating* genes stems from the following logic: the in-degree, arrival rate of the negative customers from the external system and transition probabilities of an initiating gene are all 0; that is,
  $$\begin{array}{*{20}l} D_{i}^{\; in} \; &= \; \lambda_{i}^{-} \; = \; p_{{ji}}^{+} \; = \; p_{{ji}}^{-} \; = \; 0 \;\; \text{for} \; i \in\\& \left\{ \text{Indices of initiating genes} \right\}. \end{array} $$
  In such cases, the parameters of stationary distribution in Eq. 3 are $q_{i} = \frac {\lambda _{i}^{+}}{\mu _{i}} $, resulting in $\lambda _{i}^{+} = q_{i} \times \mu _{i} $ for all *i*∈{Indices of initiating genes}. Because the numerical value of *q*_*i*_ is unknown yet, we estimate it based on the property of the geometric distribution with parameter (1−*q*_*i*_) as follows:
  16 $$\begin{array}{*{20}l} \bar{x}_{i} & = \frac{1 - \left(1 - q_{i} \right)}{1 - q_{i}} = \frac{q_{i}}{1 - q_{i}}  \\ \Rightarrow \;\;\; \bar{q}_{i} & = \frac{\bar{x}_{i}}{1 + \bar{x}_{i}}  \end{array} $$
  Equation 16 finally leads to the definition of $\lambda _{i}^{+}$ for *i*∈{Indices of initiating genes}. Note that, according to Eq. 3, $\bar {q}_{i}$ describes the estimate of the steady-state probability that there is at least one mRNA of the gene *i* [18].

Rules and assumptions of other parameters (shown in Table 2) in this research are identical to those in [18].


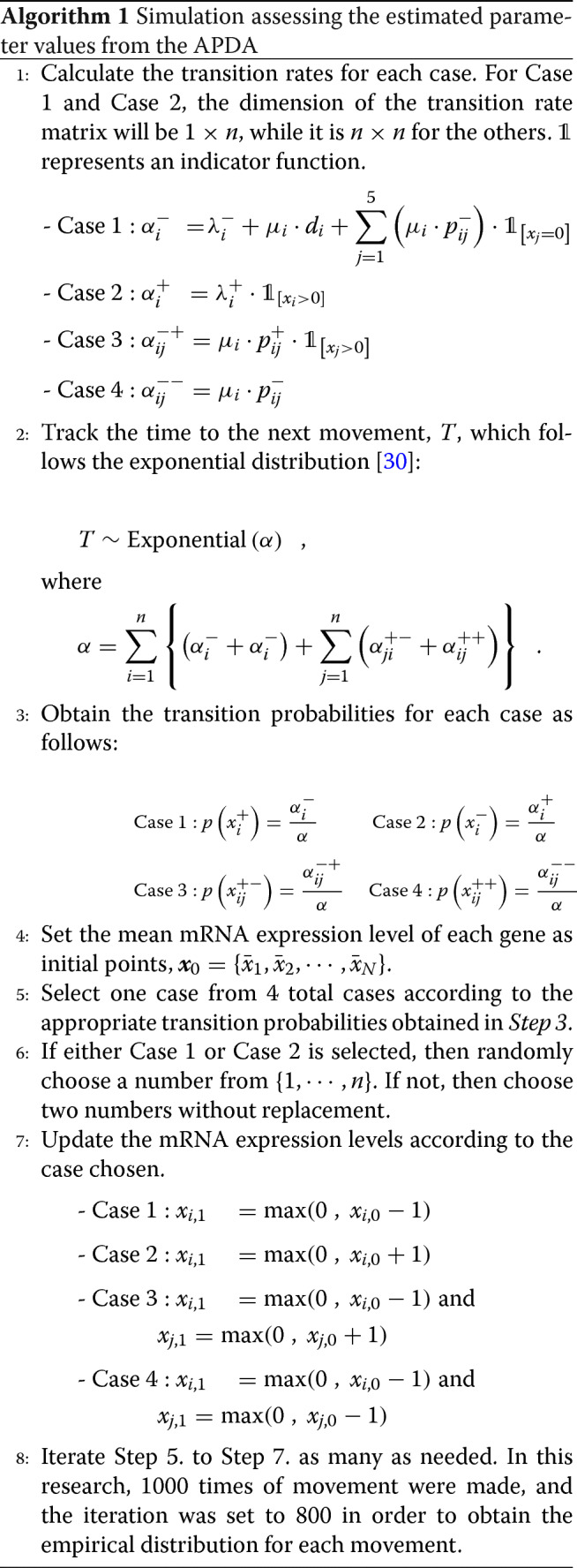


### Algorithm: simulation study

According to the global balance equation of G-Networks [23–25], a movement of mRNA expression levels of a gene in small time *Δt* is decided by one of the 4 cases as shown in Table 6.

**Table 6:** Possible movements of mRNA expression levels

Case 1	When *Genei* loses one customer
	$x_{i}^{-} = (x_{1}, \cdots, x_{i} - 1, x_{n})$
Case 2	When *Genei* gains one customer
	$x_{i}^{+} = (x_{1}, \cdots, x_{i} + 1, x_{n})$
Case 3	When *Genei* loses one customer, while *Genej* gains one
	$x_{{ij}}^{-+} = (x_{1}, \cdots, x_{i} - 1, x_{i+1}, \cdots, x_{j} + 1, x_{j+1}, \cdots, x_{n})$
Case4	When both *Genei* and *Genej* lose one customer
	$x_{{ij}}^{--} = (x_{1}, \cdots, x_{i} - 1, x_{i+1}, \cdots, x_{j} - 1, x_{j+1}, \cdots, x_{n})$

Algorithm 1 contains the details of the simulation assessing the estimated parameter values from the APDA.

## Appendix: supplementary figures/tables

**Table 7:** Correlation coefficients at ***t=0.2*** for each type of cells (normal, ALT, and telomerase-positive cells)

	Time *t*=0.2		
	Normal	ALT	Telomerase
Gene 1 and Gene 2	0.3321	0.3219	0.3414
Gene 1 and Gene 3	0.3258	0.3140	0.3317
Gene 1 and Gene 4	0.2190	0.2097	0.2046
Gene 1 and Gene 5	0.2905	0.2271	0.2234
Gene 2 and Gene 3	0.2943	0.3095	0.3055
Gene 2 and Gene 4	0.2028	0.2047	0.1951
Gene 2 and Gene 5	0.2398	0.2454	0.2260
Gene 3 and Gene 4	0.1995	0.2007	0.1936
Gene 3 and Gene 5	0.2254	0.2289	0.2188
Gene 4 and Gene 5	0.1583	0.1519	0.1396

**Table 8:** Mean mRNA expression levels (normalized and scaled) of genes in normal and telomerase-active (Telom.) cells

	Normal	Telom.	Mean diff.	*P*-value
CEBPA (Gene 1)	4.643	2.589	2.054	0.003*
E2F1 (Gene 2)	3.428	2.893	0.535	0.190
FOXM1 (Gene 3)	2.918	3.020	-0.102	0.754
c-MYC (Gene 4)	1.842	3.290	-1.448	0.000*
hTERT (Gene 5)	1.874	3.282	-1.408	0.000*

*P*-values were obtained from the Student’s t–test based on the mean differences of the expression levels. An asterisk indicates statistical significance of *p*<0.05

**Table 9:** Parameters of stationary distribution, *q*_*i*∈{1,2,⋯*N*}_ in Eq. 3, of normal and malignant (either ALT or telomerase) cells

	CEBPA	E2F1	FOXM1	c-MYC	hTERT
Normal (for ALT)	0.8253	0.7122	0.7130	0.6618	0.7269
ALT	0.7235	0.7571	0.7570	0.7637	0.7546
Normal (for telomerase)	0.8228	0.7741	0.7448	0.6481	0.6521
Telomerase	0.7478	0.7431	0.7513	0.7669	0.7429

Note that the APDA [18] differently estimates *λ*^−^ (degradation) of normal cells depending on the cell type to be compared

## Data Availability

Data used in this research can be downloaded from the National Center for Biotechnology Information Gene Expression Omnibus. Further details are in the *Data description* section.

## Abbreviations

ALT: Alternative lengthening of telomeres
SAN: Stochastic automata network
GRN: Gene regulatory network
APDA: Abnormal pathway detection algorithm
AML: Acute myeloid leukemia
ECDF: Empirical cumulative density function
CDF: Cumulative density function
